# The impact of venous resection in pancreatoduodectomy: A systematic review and meta-analysis

**DOI:** 10.1097/MD.0000000000027438

**Published:** 2021-10-08

**Authors:** João Emílio Lemos Pinheiro Filho, Francisco Tustumi, Fabricio Ferreira Coelho, Sérgio Silveira Júnior, Fernanda Cavalcanti Cabral Honório, Alexandre Cruz Henriques, André Roncon Dias, Jaques Waisberg

**Affiliations:** a Hospital Estadual Mario Covas, Santo Andre, SP, Brazil; b Universidade de São Paulo, Sao Paulo, SP, Brazil.

**Keywords:** pancreatic neoplasms, pancreatoduodenectomy, vascular surgical procedures

## Abstract

**Background:**

Vein resection pancreatoduodenectomy (VRPD) may be performed in selected pancreatic cancer patients. However, the main risks and benefits related to VRPD remain controversial.

**Objective:**

This review aimed to evaluate the risks and survival benefits that the VRPD may add when compared with standard pancreatoduodenectomy (PD).

**Methods:**

A systematic review and meta-analysis of studies comparing VRPD and PD were performed.

**Results:**

VRPD was associated with a higher risk for postoperative mortality (risk difference: −0.01; 95% confidence interval [CI] −0.02 to −0.00) and complications (risk difference: −0.05; 95% CI −0.09 to −0.01) than PD. The length of hospital stay was not different between the groups (mean difference [MD]: −0.65; 95% CI −2.11 to 0.81). In the VRPD, the operating time was 69 minutes higher on average (MD: −69.09; 95% CI −88.4 to −49.78), with a higher blood loss rate (MD: −314.04; 95% CI −423.86 to −195.22). In the overall survival evaluation, the hazard ratio for mortality during follow-up on the group of VRPD was higher compared to the PD group (hazard ratio: 1.13; 95% CI 1.03–1.23).

**Conclusion:**

VRPD is associated with a higher risk of short-term complications and mortality and a lower probability of survival than PD. Knowing the risks and potential benefits of surgery can help clinicians to properly manage pancreatic cancer patients with venous invasion. The decision for surgery with major venous resection should be shared with the patients after they are informed of the risks and prognosis.

## 1 Introduction

Pancreatic ductal adenocarcinoma is the fourth leading cause of cancer-related death in the United States.^[1]^ The worldwide total number of pancreatic cancer will increase from 62,000 to 88,000 in the next decade.^[2]^ Surgical resection is the only potentially curative treatment. Unfortunately, due to the usual late presentation, only 15% to 20% of patients are candidates for curative intent surgical intervention.^[1]^ Some patients submitted to surgery will have intraoperative diagnosis of unresectability.^[3]^ The prognosis is poor, even after complete resection. Five-year overall survival after pancreatoduodenectomy (PD) is nearly 25% to 30% for node-negative and 10% for node-positive disease.^[1]^

PD for pancreatic cancer is technically demanding, with a high risk for perioperative complications and mortality.^[4]^ In centers of excellence, morbidity rate ranges from 35% to 44%,^[5–8]^ and perioperative mortality from 2.5% to 6%.^[9–11]^

Pancreatic head cancer infiltrating vessels have historically been considered inoperable.^[12]^ Several studies have proposed en-bloc resection of the pancreas and major peripancreatic vessels to increase resectability.^[13–17]^ The massive invasion of major arteries such as the superior mesenteric artery, celiac axis, and hepatic artery are usually treated as non-curative intent, and palliation is advised.^[18]^ However, partial venous resection, such as a portal vein, superior mesenteric vein, and splenic vein, are feasible, and several centers have been performing these procedures.^[12]^

The major vessel resection in pancreatic cancer has an inherent high level of surgical technique, including microvascular anastomoses and the use of grafts.^[19]^ These difficulties impose an additional morbidity risk to the perioperative period compared with the standard PD with no vein resection.^[19]^

The aim of this review was to compare the short and long-term outcomes after standard PD and vein resection pancreatoduodenectomy (VRPD) for treatment of patients with pancreatic head ductal adenocarcinoma.

## 2 Methods

### 2.1 Database search

A systematic search was performed in PubMed, Embase, Cochrane Library Central, SciELO/LILACS, and gray literature up to April 2020. The search strategy for PubMed was ([Pancreas] OR [Pancreatic]) AND ([Neoplasm] OR [Cancer] OR [Tumor] OR [Adenocarcinoma]) AND ([Pancreaticoduodenectom∗] OR [Duodenopancreatectom∗]) AND [Mesenteric vein] OR [Vascular resection] OR [Portal vein] OR [Portal system] OR [Vein resection] OR [Vascular reconstruction] OR [Vein reconstruction]). A similar search strategy was used for other databases. The study was registered on the International Prospective Register of Systematic Reviews with registry number CRD42020201842.

Two independent authors performed literature screening. Any disagreement regarding final study inclusion was resolved by consensus. A third senior author served as the final arbiter if a consensus was not reached.

### 2.2 Study selection

Controlled clinical trials, case-control studies, and comparative cohorts were considered eligible for inclusion. Editorials, letters, conference proceedings, reviews, case reports, animal models were excluded. No restriction was set for period or language. Inclusion criteria were studies evaluating short and/or long-term outcomes after PD and VRPD (portal vein, superior mesenteric vein, or portomesenteric confluence) in adult patients (>18 years) with histologically proven adenocarcinoma of the pancreas. Exclusion criteria were patients undergoing total or distal pancreatectomy, and combined arterial resection. When more than one study with the same population was identified, only the most recent one was included.

### 2.3 Study quality assessment

Study quality was assessed using Robins-I,^[20]^ and certainty assessment was performed using GRADE.^[21]^

### 2.4 Data extraction

Full text, tables, and figures of selected studies were independently assessed by 2 researchers for data extraction including baseline characteristics: authors, year of publication, and title; patient and tumor characteristics: age, gender, serum CA19.9, tumor size, TNM stage, follow-up; type of venosus resection, and outcomes data. The following outcomes were studied in both groups (PD and VRPD): estimated blood loss, operative time, length of hospital stay, complication rate, perioperative mortality, frequency of compromised margins, and overall survival.

### 2.5 Statistical analysis

The absolute numbers for the outcome parameters were extracted and analyzed with the STATA 16.1 software (StataCorp LLC, 4905 Lakeway Drive College Station, Texas 77845-4512, USA). Continuous variables were summarized as mean difference (MD) and 95% confidence interval (95% CI). Categorical variables were summarized as risk difference (RD), or hazard ratio (HR) and 95% CI. HR and their corresponding lower and upper 95% CI limits were extracted for the individual time-to-event outcome parameters of the included studies. If the HR and their associated standard error or CI was not provided, HRs were calculated using different statistical methods based on the clinical and statistical data reported in the primary studies.^[22,23]^ A random-effects analysis model was applied to adjust for expected inter-study heterogeneity, which is more conservative when determining CI around the pooled HR.^[24]^ I^2^ statistics were applied to assess the presence of statistical heterogeneity.^[25]^ The level of significance was set at 5% (*P*-value <.05). Subgroup analysis was performed according to the neoadjuvant therapy information provided by the included studies.

## 3 Results

A total of 1184 studies were obtained using the initial search criteria. After the studies were screened, and eligibility criteria were applied, 36 studies^[26–61]^ were included in the meta-analysis (File S1, Supplemental Digital Content). Only observational studies were found. Three studies^[37,42,50]^ used neoadjuvant therapy in more than 50% of the patients and were evaluated in the subgroup analysis. No study used neoadjuvant therapy for all patients. The risk of bias and certainty assessment are shown in Files S2 and S3 Supplemental Digital Content, respectively.

### 3.1 Baseline characteristics

A total of 2986 individuals were included after study selection. The mean age across the studies was 63.8 years, with male predominance (54%). Relevant data from the included studies are shown in Table 1.

**Table 1:** The main characteristics of the included studies. Both groups “pancreatoduodenectomy associated with major venous resection” and “standard pancreatoduodenectomy” were depicted.

			Pancreatoduodenectomy associated with venous resection							Standard pancreatoduodenectomy					
Author	Follow-up (mo)	Year	N	Male (%)	Tumor size (cm)	Mean age (yr)	Vein resection	Neoadjvant therapy (%)	Serum CA 19.9	N	Male (%)	Tumor size (cm)	Mean age (yr)	Neoadjvant therapy (%)	Serum CA 19.9
FLIS	Up to 100	2016	22	41	NI	64	Portal vein, superior mesenteric vein	NI	NI	111	41	NI	66	NI	NI
HARTEL	Up to 120	2002	68	57	3.2	64	Portal vein, superior mesenteric vein, portomesenteric confluence	NI	NI	203	65	3	61	NI	NI
CARRERE	26	2006	45	71	3.18	59	Portal vein	NI	798	88	67	2.7	61	NI	947
ZHAO	18	2016	21	62	3.7	63	Portal vein, superior mesenteric vein, portomesenteric confluence	NI	NI	85	52	2.8	63	NI	NI
WELSCH	12	2016	113	50	3.6	69	Portal vein, superior mesenteric vein	14	NI	66	53	3.3	67	2	NI
ADDEO	82	2017	91	57	3	66	Portal vein, superior mesenteric vein, portomesenteric confluence	NI	NI	90	60	3.1	67	NI	NI
BRENNAN	Up to 109	1996	58	50	4	63	Portal vein	NI	NI	274	52	3.5	66	NI	NI
TAN TO CHEUNG	Up to 120	2014	32	63	3	63	Portal vein	0	NI	46	54	3	67	0	NI
CHAKRAVARTY	10	2010	12	58	3.6	62	Portal vein, portomesenteric confluence	NI	NI	75	67	3.3	63	NI	NI
WANG, WL	Up to 40	2015	42	62	NI	59	Portal vein, superior mesenteric vein	NI	941	166	69	NI	60	NI	1446
WANG, F	29	2014	64	53	4.2	66	Portal vein, superior mesenteric vein	NI	NI	58	52	3.3	67	NI	NI
TURLEY	29	2012	42	52	3.4	63	Portal vein, superior mesenteric vein	55	NI	162	50	2.6	66	45	NI
TOOMEY	Up to 150	2009	48	56	NI	67	Portal vein, superior mesenteric vein, portomesenteric confluence, splenic vein	NI	NI	172	47	NI	68	NI	NI
SHIMADA	18	2006	86	57	NI	NI	Portal vein, superior mesenteric vein	NI	NI	63	62	NI	NI	NI	NI
SGROI	Up to 96	2015	60	53	NI	64	Portal vein, superior mesenteric vein	38	NI	87	49	NI	67	9	NI
LANDI	23	2015	10	70	3.1	59	Portal vein	30	29	68	46	2.5	66	0	170
ROCH	15	2016	90	57	NI	66	Portal vein	59	NI	477	57	NI	66	4	NI
RAVIKUMAR	13	2014	230	50	3	65	Portal vein, superior mesenteric vein	NI	NI	840	56	3	66	NI	NI
POON	33	2004	12	58	3	61	Portal vein	0	NI	38	63	3.5	62	0	NI
BEANE	NI	2016	194	44	NI	65	NI	28	NI	1163	52	NI	64	8	NI
NAKAGOHRI	12	2003	33	39	3.6	58	Portal vein	NI	NI	48	71	3.6	65	NI	NI
MURAKAMI	18	2015	435	53	NI	NI	Portal vein	33	NI	502	41	NI	NI	18	NI
MENON	20	2013	18	33	4.2	67.2	Portal vein, portomesenteric confluence	44	NI	43	49	3	69	5	NI
MARTIN	24	2009	36	50	3.8	62	Portal vein, superior mesenteric vein	14	NI	34	NI	NI	NI	NI	NI
LEACH	17	1998	31	61	3.5	66	Portal vein, superior mesenteric vein, portomesenteric confluence	71	NI	44	52	3	64	55	NI
KELLY	16	2013	70	40	3.25	67	Portal vein, superior mesenteric vein, portomesenteric confluence	NI	NI	422	51	3	65	0	NI
KANEOKA	32	2009	42	57	NI	66	Portal vein, superior mesenteric vein, portomesenteric confluence, splenic vein	0	NI	42	67	NI	65	0	NI
JEONG	16	2014	46	65	3.17	60	Portal vein	0	NI	230	56	2.9	61	1	NI
HRISTOV	21	2010	20	65	2.8	63.5	Portal vein	NI	NI	140	59	3	54	NI	NI
HOWARD	13	2003	13	54	3.3	68	Portal vein	NI	NI	23	61	2.7	67	NI	NI
GONG	38	2013	119	61	4	59	Portal vein	NI	172	447	66	3	59	NI	106
DELPERO	20	2015	402	53	3.6	65	Portal vein	20	818	997	58	3	66	7	717
CHERUKURU	Up to 36	2018	26	NI	4	NI	Portal vein	19	NI	15	NI	3	NI	NI	NI
CASTLEBERRY	NI	2012	281	49	NI	65	Portal vein, superior mesenteric vein	12	NI	3301	52	NI	65	6	NI
BANZ	Up to 60	2012	51	47	3	67	Portal vein	NI	NI	275	53	3	65	NI	NI
FUHRMAN	NI	1996	23	NI	3.7	NI	Portal vein, superior mesenteric vein, portomesenteric confluence	NI	NI	36	NI	3	NI	NI	NI

NI = not informed.

### 3.2 Short-term outcomes

In the VRPD group, the mean operative time was 69 minutes higher (MD: −69.09 minutes; 95% CI −88.4 to −49.78; *P*-value: <.001; I^2^: 91.4%; certainty assessment: very low); with additional blood loss (MD: −314.04 mL; 95% CI −423.86 to −195.22; *P*-value: <.001; I^2^: 88.4%; certainty assessment: very low). VRPD was associated with higher rate of complications (RD: −0.05; 95% CI −0.09 to −0.01; *P*-value: .01; I^2^: 47.9%; certainty assessment: low), and perioperative mortality (RD: −0.01; 95% CI −0.02 to −0.00; *P*-value: .02; I^2^: 0%; certainty assessment: moderate) when compared to PD. However, the length of hospital stay was not different between the groups (MD: −0.65; 95% CI −2.11 to 0.81; *P*-value: .38; I^2^: 74.7%; certainty assessment: very low).

**Figure 1:**
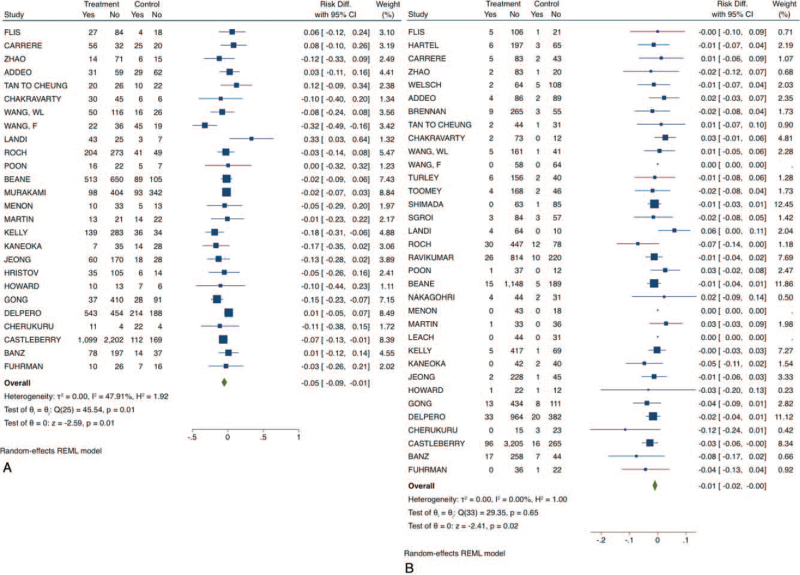
Short-term outcomes of venous resection pancreatoduodenectomy compared to standard pancreatoduodenectomy. A) Complications; B) perioperative mortality. CI = confidence interval.

**Figure 2:**
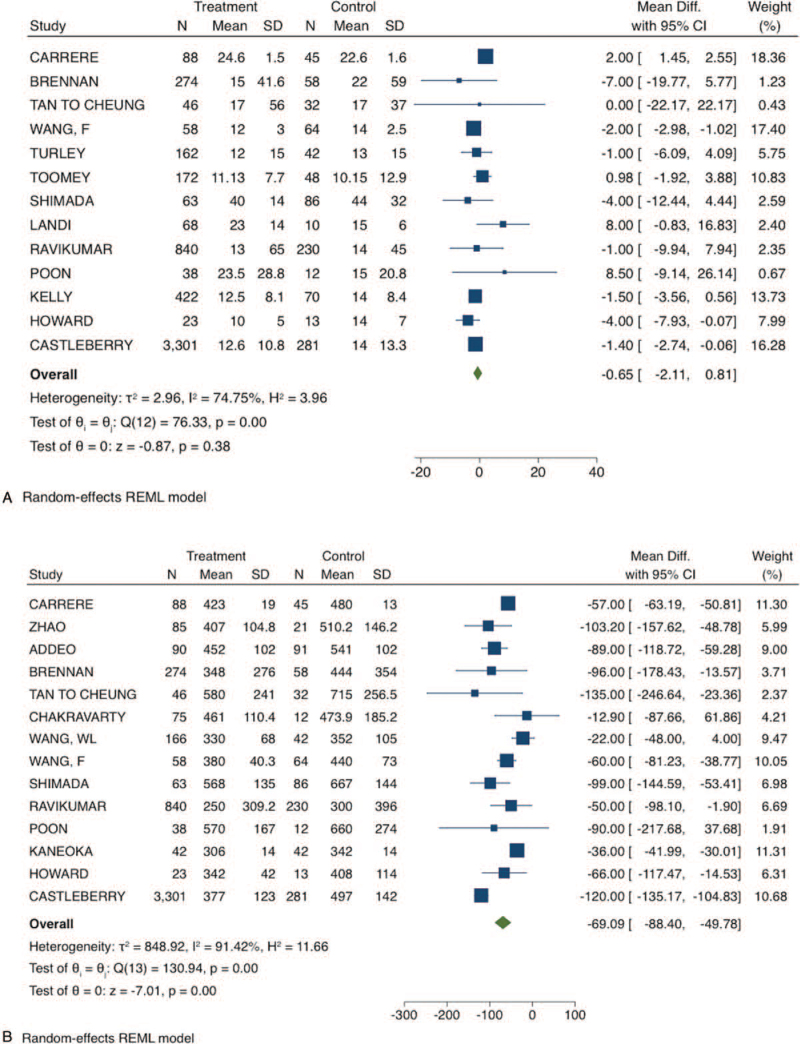
Short-term outcomes of venous resection pancreatoduodenectomy compared to standard pancreatoduodenectomy. A) Length of hospital stay; B) operative time. CI = confidence interval.

**Figure 3:**
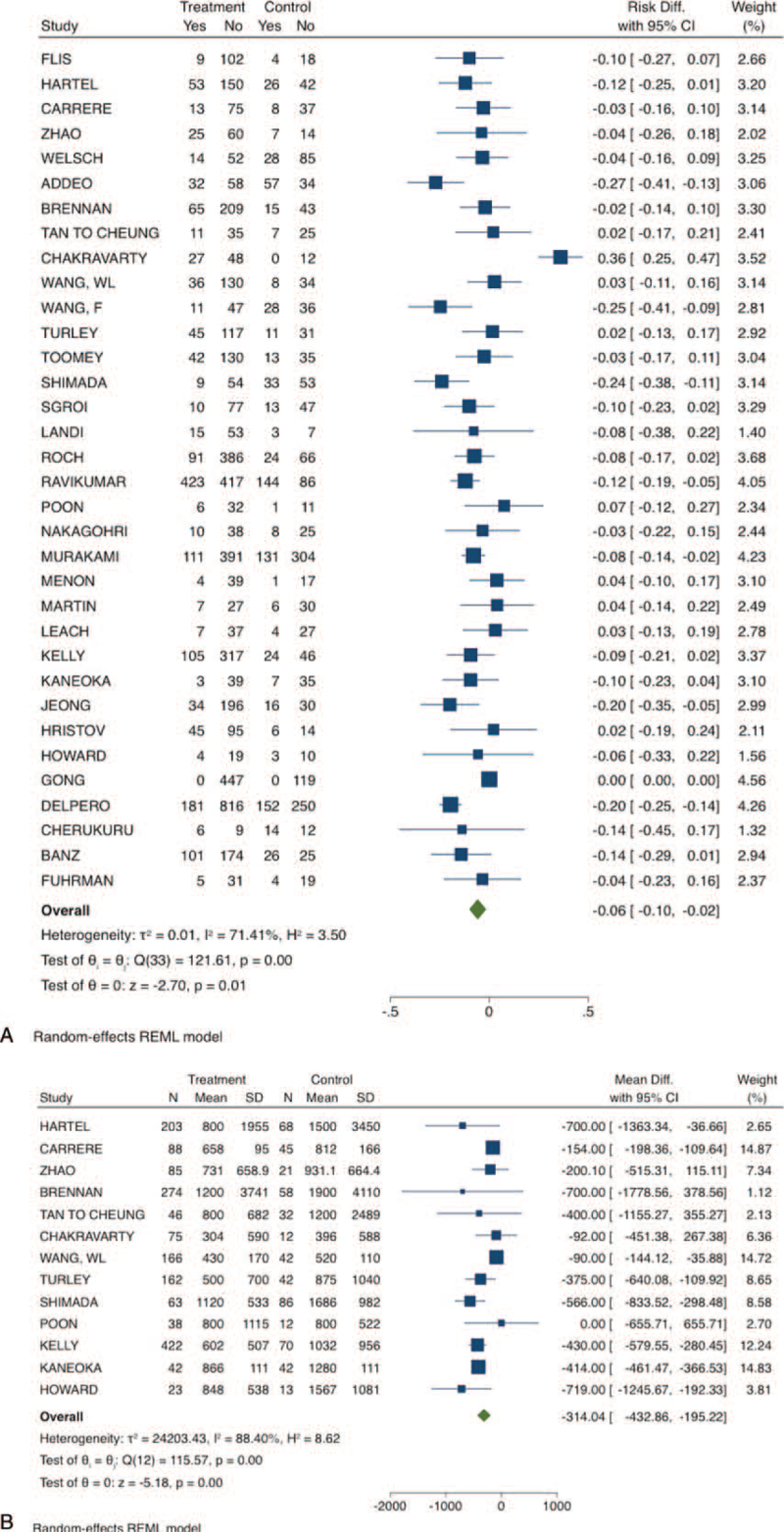
Short-term outcomes of venous resection pancreatoduodenectomy compared to standard pancreatoduodenectomy. A) Compromised margins; B) estimated blood loss. CI = confidence interval.

The rate of positive margins (R1 or R2 resection) was higher in the VRPD group (RD: −0.06; 95% CI −0.1 to −0.02; *P*-value: .01; I^2^: 74.7%; certainty assessment: very low). The summary of the short-term findings is shown on Table 2 and the forest-plots in Figures 1–3.

**Table 2:** Summary of the main short- and long-term outcomes of VRPD compared to standard PD.

Short-term outcomes				
	Measure of effect	95% CI		
Categorical variables	Risk difference	Lower	Upper	*P*-value
Postoperative complications (PD–VRPD [events])	−0.05	−0.09	−0.01	.01
Postoperative mortality (PD–VRPD [events])	−0.01	−0.02	−0	.02
Positive margins (PD–VRPD [events])	−0.06	−0.1	−0.02	.01

	Measure of effect	95% CI		
Continuous variables	Mean difference	Lower	Upper	*P*-value
Length of hospital stay (PD–VRPD [d])	−0.65	−2.11	0.81	.38
Operating time (PD–VRPD [min])	−69.09	−88.4	−49.78	<.001
Blood loss (PD–VRPD [mL])	−314.04	−423.86	−195.22	<.001

Long-term outcome				
	Measure of effect	95% CI		
	HR	Lower	Upper	*P*-value
Overall survival (VRPD/PD)	1.13	1.03	1.23	.002

CI = confidence interval, HR = hazard ratio, PD = pancreatoduodenectomy, VRPD = vein resection pancreatoduodenectomy.

In subgroup analysis including only studies that applied neoadjuvant therapy in at least 50% of the patients, no significant difference between VRPD and PD was noted in postoperative mortality (RD: −0.01; 95% CI −0.05 to 0.02; *P*-value: .52; I^2^: 16.7%), and positive margins (RD: −0.03; 95% CI −0.11 to 0.05; *P*-value: .42; I^2^: 5.3%). For the other short-term outcomes, subgroup analysis was not possible due to the lack of data in the included studies. See File S4, Supplemental Digital Content.

### 3.3 Long-term outcomes

**Figure 4:**
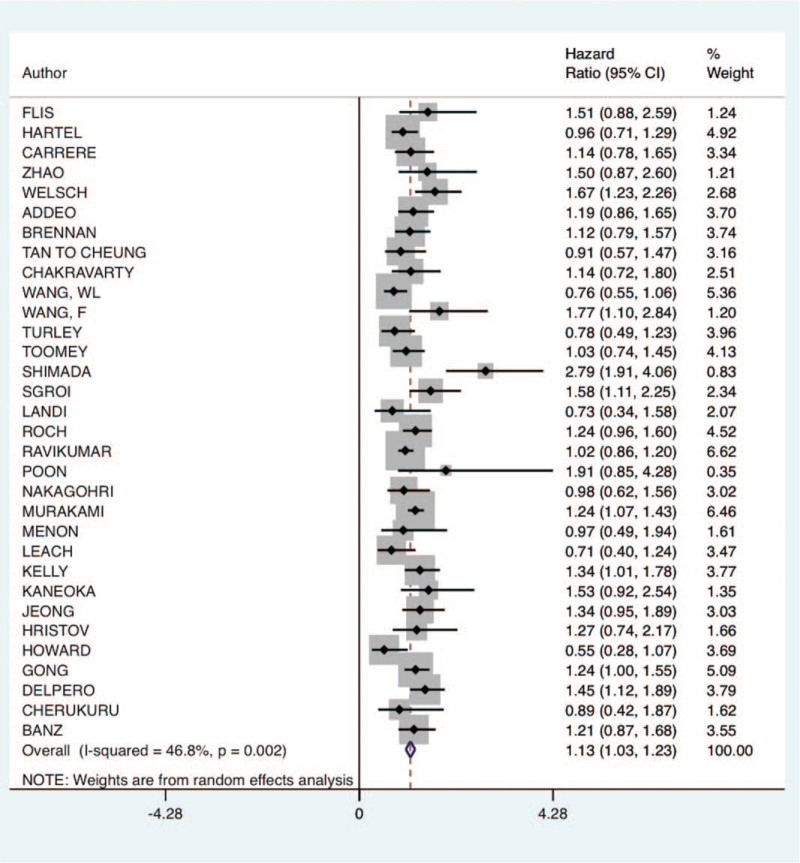
Long-term survival analysis of venous resection pancreatoduodenectomy compared to standard pancreatoduodenectomy. CI = confidence interval.

On overall survival evaluation, the hazard for mortality during follow-up on the group of VRPD was higher than the PD group (HR: 1.13; 95% CI 1.03–1.23; *P*-value: .002; I^2^: 46.8%; certainty assessment: low). See Table 2 and Figure 4. However, in the subgroup analysis, for studies that applied neoadjuvant therapy in at least 50% of the patients, there was no difference between VRPD and PD concerning long-term survival (HR: 0.93; 95% CI 0.58–1.27; *P*-value: .072; I^2^: 62%). See Supp. File 4.

## 4 Discussion

This systematic review and meta-analysis showed that the estimated blood loss, morbidity rate and perioperative mortality are higher when venous resection is performed during PD. Another worrisome finding was the worse overall survival in patients that underwent VRPD. The present systematic review gathers the best current evidence in literature of the short- and long-term outcomes of the PD with major venous resection for pancreatic cancer. These results imply that careful patient selection is imperative when major venous invasion is suspected before surgery. Any decision should be shared with the patients after they are informed of the risks. Also, VRPD should be performed only in high-volume institutions since they achieve better outcomes.^[9,62,63]^

A carefully preoperative patient selection is crucial before deciding for PD with major venous resection, since the risks are high and prognosis is often poor. Patients with poor clinical status, malnutrition, advanced age, and multiple comorbidities are at high risk for postoperative complications,^[64]^ and thus palliative interventions should be strongly considered if major vein invasion is suspected in this population.

In patients with borderline resectable pancreatic head cancer, several authors have advocated neoadjuvant therapy to increase the likelihood for R0 resection.^[65]^ In a meta-analysis, Lee et al^[65]^ showed a higher rate of R0 resection for those submitted to neoadjuvant therapy compared to upfront surgery. However, the survival benefit was only seen in those who completed the neoadjuvant therapy with subsequent resection, and the survival rate was not improved in an intent-to-treat assessment.

In our study, only 11 studies^[30,37,40,42,45,47,48,50,53,57,59]^ reported the use of neoadjuvant therapy, and none of these studies applied neoadjuvant therapy for all patients. Preoperative therapy may help by reducing tumor size, treat micrometastasis and improve patients selection for surgery.^[66,67]^ Consequently, neoadjuvant therapy has become the standard of care for patients with pancreatic cancer with major venous invasion for several authors.^[66–68]^ When overall survival was assessed including only studies that applied neoadjuvant therapy in at least 50% of the patients^[37,42,50]^ showed similarity between the VRPD and PD results, suggesting that neoadjuvant therapy may change the future long-term results of borderline resectable pancreatic head cancer.

Concerning early postoperative outcomes, besides the usual postoperative complications after PD, such as a pancreatic leak, biliary fistula, prolonged gastric emptying, and pancreatitis,^[12]^ the en-bloc venous resection may add additional risk for intra- and postoperative bleeding and vein thrombosis, all of which may explain the higher risk for early postoperative mortality in VRPD. The longer operative time in VRPD group may also explain the higher rate of perioperative complications.^[69]^

Regarding overall survival, VRPD have higher positive-margins, which per se correlate with lower cancer-specific survival. Also, perioperative blood transfusion and intraoperative blood loss have previously been independently associated with poor long-term outcomes.^[70]^ It is still unclear why intraoperative blood loss affects long-term survival, but apparently, it affects the patient's immunity reducing immune surveillance.^[70]^ Also, those with vascular invasion have a higher risk for blood circulating micrometastasis.^[71]^

Few meta-analyses have evaluated the results of VRPD compared to PD.^[72–75]^ Most included few studies, have not evaluated both early and long-term results, or have included total or distal pancreatectomy, both a source for bias, adding heterogeneity of the pooled analysis. Peng et al^[75]^ evaluated only perioperative outcomes in a meta-analysis with 30 studies, and survival analysis was not performed. Bell et al,^[72]^ in a meta-analysis, included 16 articles, and only 3 of them were used to evaluate survival. Giovinazzo et al^[73]^ included in their review 27 studies, but only 4 of them were used for 5-years survival analysis. Yu et al^[74]^ included 22 cohorts, and 12 were used for 5-years survival analysis. In all of these three meta-analyses that evaluated survival, overall survival was similar at 1 and 3-years follow-up, but at 5 years VRPD had poorer survival rate compared to PD.

This study has several limitations. All of the included studies were observational, reducing the certainty of evidence. Also, there is high heterogeneity across the studies (eg, different surgical techniques and presence of neoadjuvant therapy); there is also an undoubtable difference in institutional experience across the studies. Considering all these factors, there is considerable risk for selection bias when determining the indications for surgery. Future clinical trials with standardized treatment protocols and neoadjuvant therapy regimens are needed to evaluate PD's efficacy and safety when associated with venous resection. Also, the impact of the use of prosthesis and grafts on VRPD, the role of genomic profile sequencing, the use of nuclear medicine and spectroscopy tests, and the pattern of lymphadenectomy should be investigated in future studies.^[76–78]^

## 5 Conclusion

The rationale of venous resection is that potentially curative head pancreatic cancer treatment is only possible when negative surgical margins are obtained (R0 resection). Nonetheless, the potential benefit of this commitment to the negative margins would be the theoretical improvement in long-term survival. The current literature lacks an evidence-based review that ponders the perioperative risks and the long-term survival rates related to the major vessel resection with PD for pancreatic cancer. Knowing the risks and potential benefits of surgery can help clinicians to properly manage pancreatic cancer patients with venous invasion. The decision for surgery with major venous resection should be shared with the patients after they are informed of the risks and prognosis.

## Acknowledgments

Not applicable.

## Author contributions

**Data curation:** Fabricio Ferreira Coelho, Fernanda Cavalcanti Cabral Honório.

**Formal analysis:** Francisco Tustumi.

**Investigation:** Fabricio Ferreira Coelho.

**Methodology:** Francisco Tustumi, Sérgio Silveira Júnior, Alexandre Cruz Henriques.

**Project administration:** Joao Emilio Lemos Pinheiro Filho, Alexandre Cruz Henriques.

**Supervision:** André Roncon Dias, Jaques Waisberg.

**Validation:** Sérgio Silveira Júnior.

**Writing – original draft:** Joao Emilio Lemos Pinheiro Filho.

**Writing – review & editing:** Joao Emilio Lemos Pinheiro Filho.

## Supplementary Material

Supplemental Digital Content

## Supplementary Material

Supplemental Digital Content

## Supplementary Material

Supplemental Digital Content

## Supplementary Material

Supplemental Digital Content
