# Supplementary material for: The impact of venous resection in pancreatoduodectomy: A systematic review and meta-analysis
Source: Medicine (Baltimore). 2021 Oct 8;100(40):e27438. doi: 10.1097/MD.0000000000027438 (PMC8500612; doi:10.1097/MD.0000000000027438)
Supplement: Supplemental Digital Content [file medi-100-e27438-s001.doc]

**Supp. File 1 (Guidelines Flow Diagram).** PRISMA flow diagram.


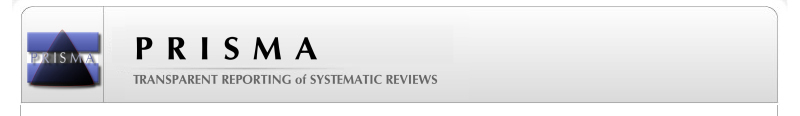
**PRISMA 2009 Flow Diagram**

**Screening**

**Included**

**Eligibility**

**Identification**

Records identified through PubMed searching
(n = 1,184)

Records identified through other bases searching
(n =399)

Records after duplicates removed
(n = 948)

Records screened
(n = 948)

Records excluded
(n = 635)

Full-text articles assessed for eligibility
(n = 72)

Full-text articles excluded

- (n = 28) Studied with no interest outcomes;
- (n = 8) Included total or distal pancreatectomy

Studies included in qualitative synthesis
(n =36)

Studies included in quantitative synthesis (meta-analysis)
(n = 36)
