# Supplementary material for: The impact of venous resection in pancreatoduodectomy: A systematic review and meta-analysis
Source: Medicine (Baltimore). 2021 Oct 8;100(40):e27438. doi: 10.1097/MD.0000000000027438 (PMC8500612; doi:10.1097/MD.0000000000027438)
Supplement: Supplemental Digital Content [file medi-100-e27438-s002.doc]

**Supp. File 2.** Risk of bias assessment (the Robins-I tool).

**Supp. Data 2.** Risk of bias assessment of included studies using ROBINS-I tool (Summary)

| The risk of bias in non-randomized studies of interventions (ROBINS-I) assessment tool for cohort-type studies | | | | | | | | |
| --- | --- | --- | --- | --- | --- | --- | --- | --- |
|  | **1. Bias due to confounding** | **2. Bias in selection of participants into the study** | **3. Bias in classification of interventions** | **4. Bias due to deviations from intended interventions** | **5. Bias due to missing data** | **6. Bias in measurement of outcomes** | **7. Bias in selection of the reported result** | **8. Overall bias** |
| FLIS | Low | Critical | Low | Low | Critical | Low | Low | Moderate |
| HARTEL | Low | Critical | Low | Low | Critical | Low | Low | Moderate |
| CARRERE | Low | Critical | Low | Low | Moderate | Low | Low | Moderate |
| ZHAO | Low | Critical | Low | Low | Moderate | Low | Low | Moderate |
| WELSCH | Low | Critical | Low | Low | Moderate | Low | Low | Moderate |
| ADDEO | Low | Critical | Low | Low | Moderate | Low | Low | Moderate |
| BRENNAN | Low | Critical | Low | Low | Critical | Low | Low | Moderate |
| TAN TO CHEUNG | Low | Critical | Low | Low | Critical | Low | Low | Moderate |
| CHAKRAVARTY | Low | Critical | Low | Low | Moderate | Low | Low | Moderate |
| WANG, WL | Low | Critical | Low | Low | Critical | Low | Low | Moderate |
| WANG, F | Low | Critical | Low | Low | Moderate | Low | Low | Moderate |
| TURLEY | Low | Critical | Low | Low | Moderate | Low | Low | Moderate |
| TOOMEY | Low | Critical | Low | Low | Critical | Low | Low | Moderate |
| SHIMADA | Low | Critical | Low | Low | Moderate | Low | Low | Moderate |
| SGROI | Low | Critical | Low | Low | Critical | Low | Low | Moderate |
| LANDI | Low | Critical | Low | Low | Moderate | Low | Low | Moderate |
| ROCH | Low | Critical | Low | Low | Moderate | Low | Low | Moderate |
| RAVIKUMAR | Low | Critical | Low | Low | Moderate | Low | Low | Moderate |
| POON | Low | Critical | Low | Low | Moderate | Low | Low | Moderate |
| BEANE | Low | Critical | Low | Low | Critical | Low | Low | Moderate |
| NAKAGOHRI | Low | Critical | Low | Low | Moderate | Low | Low | Moderate |
| MURAKAMI | Low | Critical | Low | Low | Moderate | Low | Low | Moderate |
| MENON | Low | Critical | Low | Low | Moderate | Low | Low | Moderate |
| MARTIN | Low | Critical | Low | Low | Moderate | Low | Low | Moderate |
| LEACH | Low | Critical | Low | Low | Moderate | Low | Low | Moderate |
| KELLY | Low | Critical | Low | Low | Moderate | Low | Low | Moderate |
| KANEOKA | Low | Critical | Low | Low | Moderate | Low | Low | Moderate |
| JEONG | Low | Critical | Low | Low | Moderate | Low | Low | Moderate |
| HRISTOV | Low | Critical | Low | Low | Moderate | Low | Low | Moderate |
| HOWARD | Low | Critical | Low | Low | Moderate | Low | Low | Moderate |
| GONG | Low | Critical | Low | Low | Moderate | Low | Low | Moderate |
| DELPERO | Low | Critical | Low | Low | Moderate | Low | Low | Moderate |
| CHERUKURU | Low | Critical | Low | Low | Critical | Low | Low | Moderate |
| CASTLEBERRY | Low | Critical | Low | Low | Critical | Low | Low | Moderate |
| BANZ | Low | Critical | Low | Low | Critical | Low | Low | Moderate |
| FUHRMAN | Low | Critical | Low | Low | Critical | Low | Low | Moderate |
