# Supplementary material for: The impact of venous resection in pancreatoduodectomy: A systematic review and meta-analysis
Source: Medicine (Baltimore). 2021 Oct 8;100(40):e27438. doi: 10.1097/MD.0000000000027438 (PMC8500612; doi:10.1097/MD.0000000000027438)
Supplement: Supplemental Digital Content [file medi-100-e27438-s003.doc]

**Supp. File 3.** Certainty assessment (GRADE).

| **Certainty assessment** | | | | | | |
| --- | --- | --- | --- | --- | --- | --- |
| **Participants  (studies)** | **Risk of bias** | **Inconsistency** | **Indirectness** | **Imprecision** | **Publication bias** | **Overall certainty of evidence** |
|
| **Perioperative complications** | | | | | | |
| 11189 (26 observational studies) | serious ^a^ | serious ^b^ | not serious | not serious | none | ⨁⨁◯◯ LOW |
| **Perioperative mortality** | | | | | | |
| 12820 (34 observational studies) | serious ^a^ | not serious | not serious | not serious | none | ⨁⨁⨁◯ MODERATE |
| **Positive margins** | | | | | | |
| 8978 (34 observational studies) | serious ^a^ | very serious ^c^ | not serious | not serious | none | ⨁◯◯◯ VERY LOW |
| **Length of hospital stay** | | | | | | |
| 6546 (13 observational studies) | serious ^a^ | very serious ^c^ | not serious | not serious | none | ⨁◯◯◯ VERY LOW |
| **Operative time** | | | | | | |
| 6085 (14 observational studies) | serious ^a^ | very serious ^c^ | not serious | not serious | none | ⨁◯◯◯ VERY LOW |
| **Estimated blood loss** | | | | | | |
| 2230 (13 observational studies) | serious ^a^ | very serious ^c^ | not serious | not serious | none | ⨁◯◯◯ VERY LOW |
| **Overall survival** | | | | | | |
| 8849 (32 observational studies) | serious ^a^ | serious ^b^ | not serious | not serious | none | ⨁⨁◯◯ LOW |

#### Explanations

a. Risk of selection bias

b. Heterogeneity > 30%

c. Heterogeneity > 50%
