# Supplementary material for: The impact of venous resection in pancreatoduodectomy: A systematic review and meta-analysis
Source: Medicine (Baltimore). 2021 Oct 8;100(40):e27438. doi: 10.1097/MD.0000000000027438 (PMC8500612; doi:10.1097/MD.0000000000027438)
Supplement: Supplemental Digital Content [file medi-100-e27438-s004.doc]

| **Short-term outcomes** | | | | |
| --- | --- | --- | --- | --- |
|  | **Measure of effect** | **95% IC** | | **p-value** |
| **Categorical variables** | **Risk difference** | **Lower** | **Upper** |  |
| Postoperative mortality (PD - VRPD [events]) | -0.01 | -0.05 | 0.02 | 0.52 |
| Positive margins (PD - VRPD [events]) | -0.03 | -0.11 | 0.05 | 0.42 |
| **Long-term outcome** | | | | |
|  | **Measure of effect** | **95% IC** | | **p-value** |
|  | **HR** | **Lower** | **Upper** |  |
| Overall survival (VRPD/PD) | 0.93 | 0.58 | 1.27 | 0.07 |

**Supp. File 4.** Subgroup analysis. Only studies with at least 50% of the patients treated with neoadjuvant therapy were included in this analysis. Summary of the main short- and long-term outcomes of venous resection pancreatoduodenectomy (VRPD) compared to standard pancreatoduodenectomy (PD).
